# Identification of a New HCV Subtype 6xg Among Injection Drug Users in Kachin, Myanmar

**DOI:** 10.3389/fmicb.2019.00814

**Published:** 2019-04-18

**Authors:** Mei Ye, Xin Chen, Yu Wang, Lin Duo, Chiyu Zhang, Yong-Tang Zheng

**Affiliations:** ^1^Key Laboratory of Animal Models and Human Disease Mechanisms of the Chinese Academy of Sciences, The National Kunming High Level Biosafety Research Center for Nonhuman Primate, KIZ-CUHK Joint Laboratory of Bioresources and Molecular Research in Common Diseases, Kunming Institute of Zoology, Chinese Academy of Sciences, Kunming, China; ^2^Kunming College of Life Science, University of Chinese Academy of Sciences, Kunming, China; ^3^Department of Pathogenic Biology, School of Basic Medical Sciences, Gannan Medical University, Ganzhou, China; ^4^KIZ-SU Joint Laboratory of Animal Model and Drug Development, College of Pharmaceutical Sciences, Soochow University, Suzhou, China; ^5^Yunnan Fuwai Cardiovascular Hospital, Kunming, China; ^6^Pathogen Discovery and Big Data Center, CAS Key Laboratory of Molecular Virology and Immunology, Institut Pasteur of Shanghai, Chinese Academy of Sciences, Shanghai, China

**Keywords:** hepatitis C virus, injection drug users, subtype, HCV 6xg, Myanmar, Yunnan

## Abstract

Characterizing hepatitis C virus (HCV) genetic diversity not only allows us to trace its origin and evolutionary history, but also provides valuable insights into diagnosis, prevention and therapy of HCV infection. Although eight HCV genotypes and 86 subtypes have been classified, there are still some HCV variants that need to be assigned. The genotype 6 is the most diverse HCV genotype and mainly prevalent in Southeast Asia. In this study, we identified a new HCV subtype 6xg from injection drug users (IDUs) in Kachin, Myanmar. A distinctive feature of 6xg from other subtypes of the genotype 6 was a Lys insertion in NS5A gene, which changes the RRKR/K motif into RRKKR/K. Bayesian analyses showed that HCV 6xg originated during 1984–1988, and experienced a rapid population expansion during 2005–2009. We characterized HCV subtype profile among IDUs in this region, and detected six HCV subtypes, including 1a (12.0%), 3a (12.0%), 3b (24.0%), 6n (16.0%), 6xa (20.0%), and 6xg (12.0%). Importantly, we found that HCV subtype distribution in Kachin was very similar to that in Dehong prefecture of Yunnan, but very distinct from those in other regions of Myanmar and Yunnan, indicating that the China–Myanmar border region shared a unique HCV subtype pattern. The appearance of 6xg and the unique HCV subtype profile among IDUs in the China–Myanmar border region have significant epidemiological and public health implications.

## Introduction

Despite living in the era of high-efficiency antiviral treatments (Sofosbuvir/Velpatasvir/Voxilaprevir: Vosevi) (Heo and Deeks, 2018), hepatitis C virus (HCV) infection is still a major global health problem especially for the developing world (Luhmann et al., 2015; Hlaing et al., 2017; WHO, 2017). There are about 71 million people living with chronic HCV infection including about 1.75 million new HCV infections worldwide and about 399,000 deaths from HCV-related cirrhosis and hepatocellular carcinoma per year (WHO, 2017).

Hepatitis C virus is a blood-borne RNA virus that is primarily transmitted through risk factors of injection drug use (IDU), blood transfusion, and sexual contact (Degenhardt et al., 2017). IDUs are the most high-risk population for HCV infection and have very high HCV prevalence (Degenhardt et al., 2017). As the most main drug-producing and drug-trafficking area during the second half of the last century, the“Golden triangle” and surrounding Southeast Asian countries/regions were worst affected by drugs and some IDU-associated infectious diseases, such as HCV and HIV-1 (Zhang et al., 2002; Zhou et al., 2011, 2012). Around 26% of the global HCV infections among IDUs was estimated to occur in Southeast Asia (Degenhardt et al., 2017). The China–Myanmar border region is one of the regions with high HCV prevalence among IDUs (Zhou et al., 2011, 2012; Li et al., 2014). Our previous studies showed that HCV prevalence among IDUs along the China–Myanmar border was over 69% on the Yunnan side, and 48.1% on the Myanmar side (Zhou et al., 2011, 2012). A study conducted in 2007 reported a much higher HCV prevalence (66.3–93.5%) among IDUs in Myanmar (Thu et al., 2008). Furthermore, a national survey carried out in 2015 revealed that HCV prevalence among the general population was 2.7% in Myanmar, which was much higher than the global prevalence rates of 1% (Lwin et al., 2017; Ministry of Health and Sports Myanmar, 2017; WHO, 2017). These indicated a very severe HCV epidemic among IDUs in Myanmar and the China–Myanmar border region.

**FIGURE 1:**
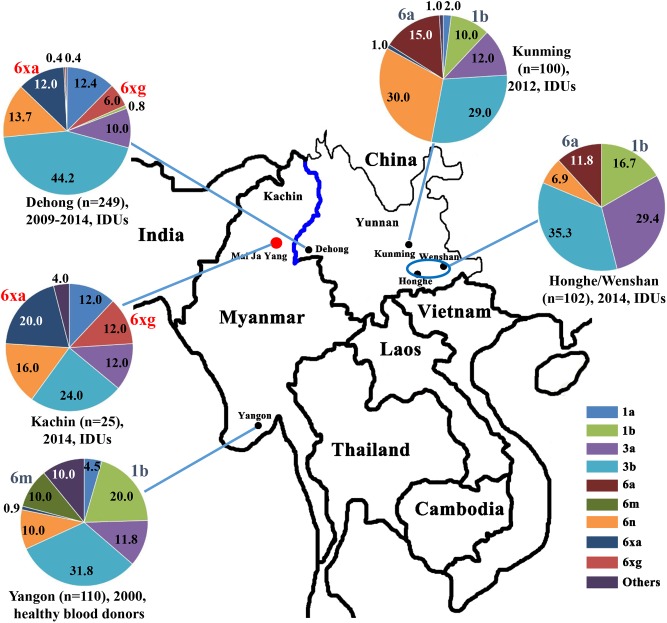
Geographical location and HCV subtype distribution among IDUs in Kachin, Myanmar. The data on HCV subtype profiles in Dehong, Kunming, Honghe and Wenshan, and Yangon were retrieved from previous studies (Shinji et al., 2004; Zhang et al., 2013; Chen et al., 2015; Wan et al., 2016).

Hepatitis C virus belongs to the *Hepacivirus* genus of the *Flaviviridae* family and has a high genetic diversity (Webster et al., 2015). Currently, HCV is classified into seven genotypes, six of which are further divided into some subtypes (Smith et al., 2014). As of June 2017, a total of 86 HCV subtypes had been confirmed by the International Committee on Taxonomy of Viruses^1^. The genetic distances between HCV genotypes are approximately 30–35% in genome level, and the distances between subtypes are 15–20% (Smith et al., 2014). There were some HCV variants that meet the threshold, but need to be assigned as new subtypes (Lu et al., 2006, 2013; Hubschen et al., 2011; Wang et al., 2013; Li et al., 2015; Wan et al., 2016). Recently, the eighth HCV genotype was identified from four patients in India (Borgia et al., 2018). In our previous study, we characterized HCV diversity among IDUs in Dehong prefecture of Yunnan, bordering with Myanmar, and found four special HCV variants from the Burmese IDUs who lived or stayed in Yunnan (Burmese IDUs staying in Yunnan), which formed an independent clade and diverged from the subtype 6n in the C/E2 and NS5B genomic regions (Wan et al., 2016). The finding suggested that these variants belong to a potential new HCV subtype. However, because of the lack of the full-length genomic sequences, we were unable to define them as a new HCV subtype. Furthermore, Burmese IDUs staying in Yunnan appeared to be relatively separated from the local IDUs in IDU behavior (Wan et al., 2016), suggesting that they acquired infection with the new variants in Myanmar. To further identify these variants and trace their origin, we performed a HCV molecular epidemiological investigation among IDUs in Kachin, Myanmar, which borders with Dehong prefecture of Yunnan (Figure 1), and identified these variants as a new HCV subtype 6xg by full-length genomic sequence analyses.

## Materials and Methods

### Ethics Approval and Study Design

The study was approved by the Ethics Committee of Kunming Institute of Zoology, Chinese Academy of Sciences (approval number: SWYX-2013023; date: September 6, 2013). Written informed consents were obtained from each participant prior to the study.

With the support of HIV/AIDS Asia Regional Program Yunnan Management Office, a cross-sectional survey was carried out in detoxification centers in the Mai Ja Yang region of Kachin State, Myanmar, 2014 (Figure 1). Socio-demographic information including gender, age, occupation, education level, ethnicity, marital status, cross-border behavior and duration of drug abuse was collected by trained interviewers with a standard questionnaire. About 5 milliliters of whole blood samples were collected from each participant using vacuous ethylenediaminetetraacetic acid dipotassium salt (EDTA-2K) tubes. Plasma was separated by centrifugation and then stored at -80°C freezer until use.

### HCV Testing

The HCV serostatus was determined with enzyme-linked immunosorbent assay testing kit for IgG antibodies against HCV (Beijing Wantai Biological Pharmacy Enterprise Co., Ltd., Beijing, China), according to the manufacturer’s instructions.

### PCR Amplifications

Viral RNA was extracted from 200 μl of plasma using the High Pure Viral RNA Kit (Roche Diagnostics Ltd., Mannheim, 11858882001). Reverse transcription was performed using PrimeScript II 1st Strand cDNA Synthesis Kit (TaKaRa Biomedical Technology Co., Ltd., Beijing, 6210A). The cDNA was amplified with a nested-PCR using TransTaq DNA Polymerase High Fidelity kit (Beijing TransGen Biotech Co., Ltd., AP131-13). Partial HCV fragments in C/E2 (H77: 849-2152) and NS5B (H77: 8266–9303) regions were amplified using the primers described previously (Wan et al., 2016). In order to obtain the near full-length genome of HCV (H77: 18-9371), we initially attempted to amplify the full-length genome (>5000 bp) using long-range RT-nested PCR with various primer sets, but failed in all these amplifications. Therefore, 10 overlapping HCV genomic fragments were amplified using RT-nested PCR. The information of all primers used in this study is shown in Supplementary Table S1. The PCR products were purified using a Gel Extraction Kit (Bioteke Corporation, DP1503) in accordance with the manufacturer’s protocol, and subjected to Sanger sequencing with an ABI PRISM 377XL DNA sequencer (Applied Biosystems, Carlsbad, CA, United States).

### Phylogenetic Analyses

The obtained sequences were aligned with HCV subtype reference sequences using HCVAlign^2^ and then were manually edited using Bioedit v7.0. The best substitution model for phylogenetic analyses was inferred using Jmodeltest v2.1.7. The general time reversible nucleotide substitution model plus a gamma distribution among site rate heterogeneity model was the best substitution model for all sequence sets. Maximum likelihood (ML) trees were constructed using MEGA 7.0 with 1000 bootstrap replications. All ML phylogenetic trees were rooted with HCV 7a as an out-group. Reference sequences were downloaded from the Los Alamos National Laboratory HCV sequence database and International Committee on Taxonomy of Viruses. Pairwise nucleotide and amino acid similarities were calculated using software BioEdit v7.0. The recombination analysis was performed with SimPlot 3.5.1 software.

In order to estimate the phylogeny of HCV genotype 6 strains among patients in the China–Myanmar border region, all available E1/E2 (H77: 933-2048) and NS5B sequences (H77: 8373-9215) of HCV genotype 6 were downloaded from GenBank. The E1/E2 dataset covers 93 sequences from Dehong (*n* = 90) and Lincang (*n* = 3), Yunnan province. The NS5B dataset includes 89 sequences from Dehong (*n* = 86) and Lincang (*n* = 3). These sequences were subjected to the phylogenetic analyses with the sequences obtained in this study.

### Bayesian Phylogenetic Analysis

To explore the evolutionary history of HCV genotype 6 in the China–Myanmar border region, the E1/E2 and NS5B sequences with known sampling years and sampling locations were subjected to the Bayesian analysis. The time to the most recent common ancestor (tMRCA) was inferred using the Markov Chain Monte Carlo (MCMC) algorithm as implemented in BEAST v1.10.0. Maximum clade credibility (MCC) trees were constructed under an uncorrelated relaxed lognormal molecular clock model, the general time reversible nucleotide substitution model plus a gamma distribution among site rate heterogeneity model and a constant population size coalescent model by BEAST v1.10.0. Each MCMC was run for 200 million generations and sampled every 20000 generations. Convergence was assessed on the basis of the effective sampling size (ESS) after a 25% burn-in using Tracer v1.7.1. Only ESS values > 200 were accepted. All of these trees were viewed and edited using FigTree v1.4.3. Population dynamics were constructed under a Bayesian skyline plot coalescent tree prior and a piecewise-linear skyline model with 10 groups by BEAST v1.10.0. The Bayesian skyline plot was reconstructed using Tracer v1.7.1.

### Statistical Analysis

The statistical analysis was performed by statistical software Statistical Package for Social Sciences (version 19.0; SPSS, Inc., Chicago, IL, United States). The normality of distribution of continuous variable (age) was tested by one-sample Kolmogorov–Smirnov test. Continuous variable with normal distribution was expressed as mean ± standard deviation; non-normal variable was shown as median ± interquartile range. Categorical variables were compared using Chi-square test. A value of *P* < 0.05 was considered statistically significant.

### GenBank Accession Numbers

The nucleotide sequences reported in this study have been submitted to GenBank with accession numbers MH458952–MH458976 for C/E2, MH458977–MH459000 for NS5B and MH492360–MH492362 for full-length genome.

## Results

### Social-Demographic Characteristics of IDUs in Kachin State, Myanmar

A total of 100 IDUs were recruited from Kachin State, Myanmar (Table 1). The participants were at a mean age of 37.3 ± 13.0 years old. The majority of them were male (88.0%) and farmers (86.0%). Most IDUs were married (56.6%), were Jingpo ethnicity (55.6%), and completed their primary school or had higher level education (77.0%). Importantly, 79.6% of them had a history of cross-border migration between Myanmar and China.

**Table 1:** Demographic characteristics of study subjects.

Variable		Total		HCV seropositive		*p*-value
			
		*N*	%	*n*	%	
Gender						0.851
	Male	88	88.0	36	40.9	
	Female	12	12.0	4	33.3	
Age						0.136
	≤25	20	20.0	7	35.0	
	26–35	29	29.0	11	37.9	
	36–45	28	28.0	16	57.1	
	≥46	23	23.0	6	26.1	
Occupation						0.724
	Farmer	86	86.0	35	40.7	
	Others^a^	14	14.0	5	35.7	
Education						0.140
	None	23	23.0	7	30.4	
	Primary school	43	43.0	22	51.2	
	Junior school or above	34	34.0	11	32.4	
Ethnicity^b^						0.184
	Jingpo	55	55.6	19	34.5	
	Others^c^	44	44.4	21	47.7	
Marital status^b^						0.214
	Unmarried	38	38.4	16	42.1	
	Married	56	56.6	24	42.9	
	Divorced or widowed	5	5.1	0	0	
Cross-border^b^						0.107
	Yes	78	79.6	35	44.9	
	No	20	20.4	5	25.0	
Duration of drug abuse^b^						0.027
	≤5 years	53	54.6	16	30.2	
	>5 years	44	45.4	23	52.3	


*^*a*^Driver, Worker, Businessman, Jobless. ^*b*^The information of questionnaires was incomplete. ^*c*^Dai, Mian, Lisu, Han, Achang, Deang, Dong, Yi.*

The HCV seroprevalence of this cohort was 40.0% (40/100). HCV prevalence was significantly associated with duration of drug use with higher positive rate among IDUs with longer duration (over 5 years) (52.3%) than those among IDUs with short duration of less than 5 years (30.2%) (*P* = 0.027), but not with gender, age, occupation, educational level, ethnicity, cross-border migration behavior, and marital status (*P* > 0.05) (Table 1).

### HCV Genotyping of the Sequences From IDUs in Kachin State, Myanmar

**FIGURE 2:**
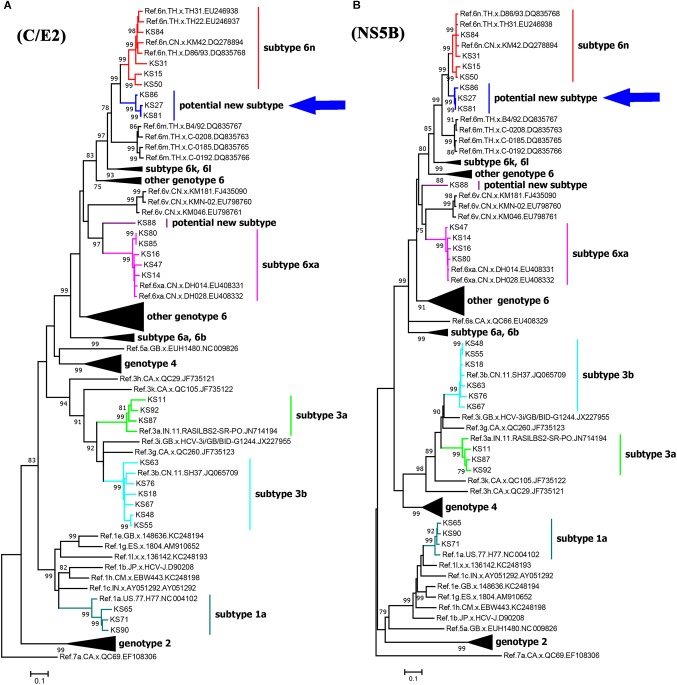
Maximum likelihood trees of C/E2 **(A)** and NS5B **(B)** fragments of HCV strains in Kachin, Myanmar. The trees were constructed using MEGA 7.0 with ML method under the “GTR+I+G” model. The reliability of interior branches in the trees was evaluated by the bootstrap method with 1000 replications. Only bootstrap values over 75% are shown. The clade of the potential new HCV subtype is highlighted by an arrow.

We successfully amplified and sequenced 25 C/E2 and 24 NS5B fragments from 25 of 40 HCV positive samples. There were 24 samples with both C/E2 and NS5B sequences, and one sample with only C/E2 sequence. HCV subtypes were determined based on the C/E2 and NS5B ML trees (Figure 2). Both ML trees showed completely consistent subtyping results for 24 samples with both C/E2 and NS5B sequences. Except four sequences (KS27, KS81, KS86, and KS88), all other sequences were able to be assigned to known HCV subtypes (Figure 2). Five HCV subtypes 1a, 3a, 3b, 6n, and 6xa (previously known as 6u) were found among this cohort, and subtype 3b appeared to be the most predominant HCV genotype (24.0%), followed by subtypes 6xa (20.0%), 6n (16.0%), 1a (12.0%), and 3a (12.0%) (Table 2). Among four unassigned sequences, three (KS27, KS81, and KS86) clustered together, forming an independent clade between the clades of subtypes 6n and 6m (Figure 2). The phylogenetic position of the clade in the trees was similar to the clade previously described (Wan et al., 2016), suggesting that these variants in this study and previous study may belong to a new subtype.

**Table 2:** The genotypes distribution of HCV among IDUs in Kachin, Myanmar.

Genomic fragment	HCV subtype n (%)						Total (n)
		
	1a	3a	3b	6n	6xa	Potential new subtypes	
C/E2	3 (12.0%)	3 (12.0%)	6 (24.0%)	4 (16.0%)	5 (20.0%)	4 (16.0%)	25
NS5B	3 (12.5%)	3 (12.5%)	6 (25.0%)	4 (16.7%)	4 (16.7%)	4 (16.7%)	24
C/E2 and or NS5B	3 (12.0%)	3 (12.0%)	6 (24.0%)	4 (16.0%)	5 (20.0%)	4 (16.0%)	25


### Identification of the HCV New Subtype 6xg

To identify and confirm the new HCV subtype, we obtained the near full-length genomic sequences of the three isolates KS27, KS81, and KS86 by amplifying and sequencing 10 overlapping HCV genomic fragments. Their genomes were composed of 9318 nt, including the 5′UTR (nt 1–272) and a single open reading frame (nt 273–9318) encoding a polyprotein precursor of 3015 amino acids. The protein coding region consisted of the core (573 nt/191 aa), E1 (576 nt/192 aa), E2 (1092 nt/364 aa), P7 (189 nt/63 aa), NS2 (651 nt/217 aa), NS3 (1893 nt/631 aa), NS4A (162 nt/54 aa), NS4B (783 nt/261 aa), NS5A (1356 nt/452 aa) and NS5B (1771 nt/590 aa) (Supplementary Table S2).

**FIGURE 3:**
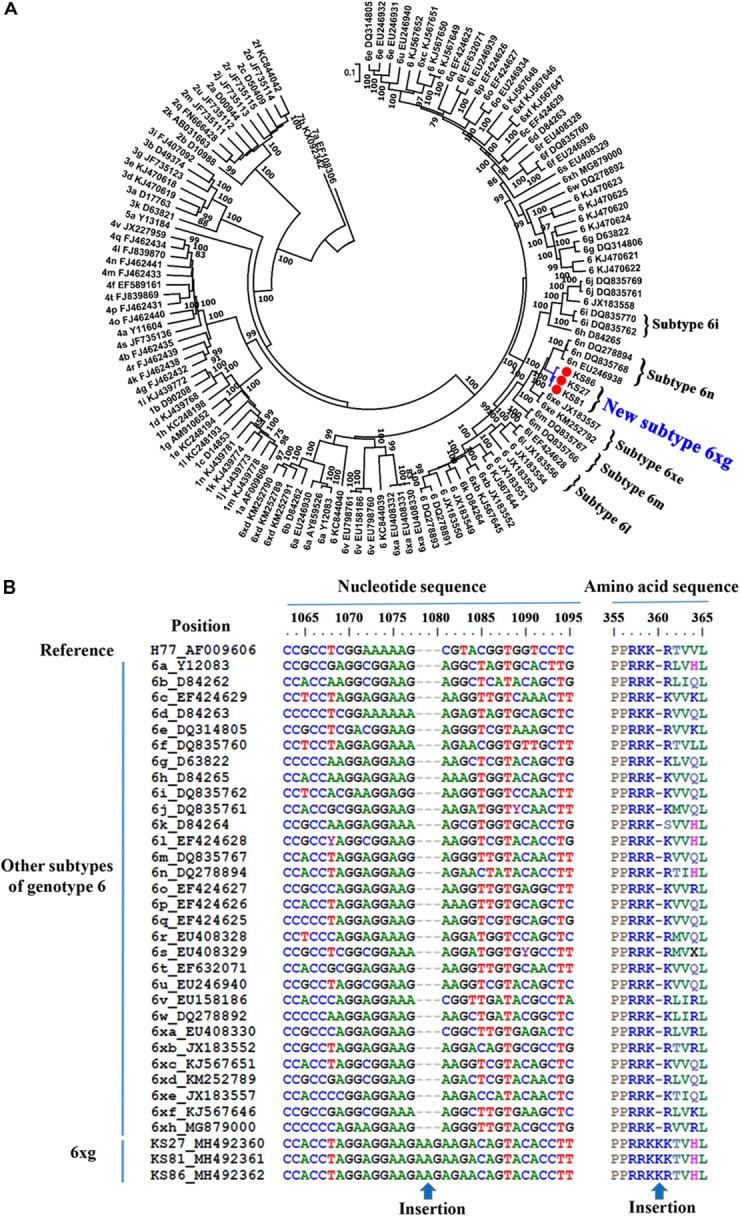
Identification of the new HCV subtype 6xg. **(A)** ML tree of 3 representative full-length genomic sequences. **(B)** NS5A sequence feature of HCV subtype 6xg.

Phylogenetic analysis of the full-length genomic sequences showed that the three strains closely clustered together, forming an independent clade with 100% bootstrap value support (Figure 3A). The clade was clustered with the clade of subtype 6n, indicating an early divergence from subtype 6n. Sequence analyses showed that the three strains had nucleotide similarities of 96.1–99.3% each other, and average similarities of 71.8–85.6% with other subtypes of the genotype 6 (Supplementary Table S3). In addition, no recombination breakpoint was detected within the three sequences. Because the sequences that were obtained from three epidemiologically unrelated individuals met the criteria of a new HCV subtype, they were assigned as new HCV subtype 6xg in alphabetical order. Compared to other subtypes of HCV genotype 6, the 6xg strains had a one-amino acid (Lys) insertion in the RRKR/K motif of NS5A (Figure 3B).

### Epidemiological Distribution of HCV Subtype 6xg in the China–Myanmar Border Region

**FIGURE 4:**
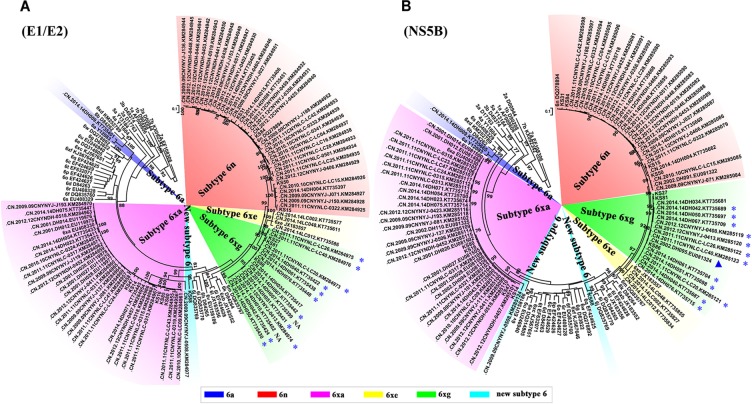
ML trees of E1/E2 **(A)** and NS5B **(B)** sequences using hit sequences. The hit sequences were obtained by HCV BLAST with HCV 6xg strains as query set. The clades of subtypes 6xg, 6xe, and 6n are highlighted by green, yellow, and red shadows, respectively. The sequences from the Burmese IDUs staying in Yunnan are highlighted by blue stars, and the strain isolated in 2002 is highlighted by a blue triangle. NA indicates that the nationality of IDUs is not available.

To explore the prevalence of the new HCV subtype 6xg in the China–Myanmar border region, we performed HCV BLAST using 6xg reference sequences obtained in this study as the query set. The top hit sequences were subjected to further phylogenetic analyses. Except the three reference sequences, 16 additional E1E2 and/or NS5B sequences were found to cluster within the clade of the subtype 6xg. The 16 strains were mainly isolated from Dehong prefecture (including Longchuan and Yingjiang counties), including 12 strains previously isolated from Burmese IDUs staying in Yunnan) (Figure 4) (Xia et al., 2008; Chen et al., 2015; Wan et al., 2016). The earliest strain of the subtype 6xg was traced to a sample (DH095) in 2002 (Xia et al., 2008), which belongs to subtype 6xg at least in NS5B region (Figure 4B). These results indicate that HCV subtype 6xg has had a regional epidemic in the China–Myanmar border region since 2002.

We then compared the subtype distributions among Kachin, Dehong, Kunming, Honghe and Wenshan, and Yangon (Figure 1). We found that HCV subtype pattern in Kachin was very similar to that in Dehong, but very distinct from those in other regions. Apart from 6xg, HCV 6xa was also mainly prevalent in Kachin and Dehong, but rare in other regions. In contrast, HCV subtypes 6m prevalent in Yangon, and 6a prevalent in Kunming, Honghe and Wenshan, were rarely detected in Kachin and Dehong. Furthermore, the most common subtype 1b was also rarely detected in Kachin and Dehong. These results indicated that the China–Myanmar border region had a different HCV subtype pattern from other regions (Figure 1).

### Evolutionary History of HCV Subtype 6xg

**FIGURE 5:**
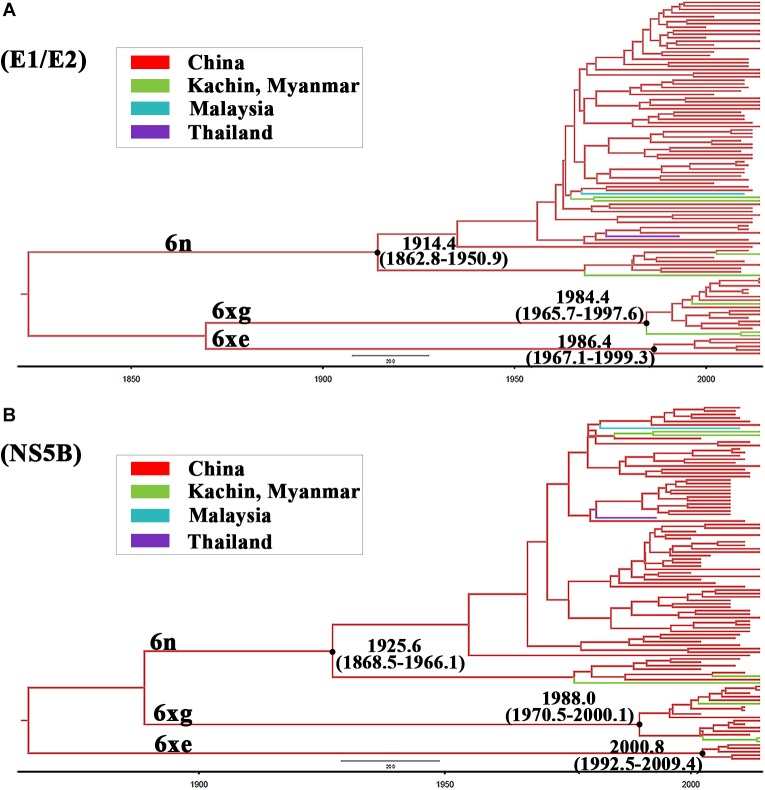
The maximum clade credibility trees of E1/E2 **(A)** and NS5B **(B)** fragments. The MCC trees were obtained by Bayesian MCMC analysis based on partial E1/E2 and NS5B sequences. The nodes of HCV subtype 6n, 6xe, and 6xg clades are shown using black dots. The node ages with 95% confidence interval are shown beside the nodes.

**FIGURE 6:**
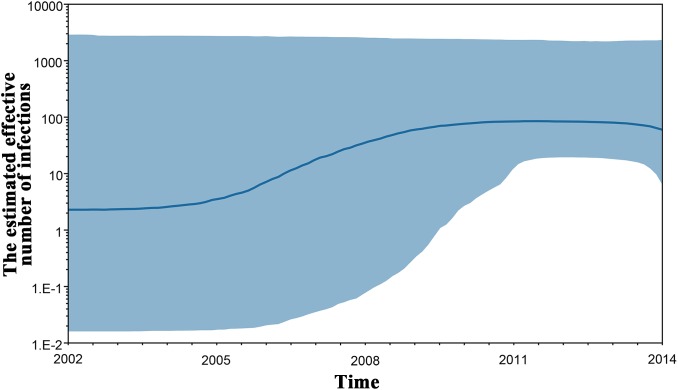
Bayesian skyline plot estimated the past population dynamics of HCV 6xg among IDUs. The *y*-axis represents the estimates of the effective number of HCV 6xg strains and the *x*-axis represents time. The solid line represents the median estimate and the shaded area represents the 95% confidence intervals.

To investigate the origin time of the new HCV subtype 6xg, Bayesian phylogenetic analyses were performed. The MCC trees showed consistent topological structures with the ML trees (Figure 3–5). The tMRCAs of the subtype 6xg were inferred to be 1984.4 (95% CI: 1965.7–1997.6) and 1988.0 (95% CI: 1970.5–2000.1) based on E1E2 and NS5B genomic regions, respectively (Figure 5), suggesting that subtype 6xg originated during 1984–1988. The origin times of two genetically related subtypes 6n and 6xe were inferred to be 1914–1925 and 1986–2000, respectively. We then simulated the population dynamics of the subtype 6xg by Bayesian skyline plot analysis of NS5B sequences. A fast exponential growth of the subtype 6xg was observed during 2005–2009, followed by a stable population size (Figure 6).

## Discussion

Hepatitis C virus genetic diversity influences its biology (e.g., infectivity, transmissibility and immunogenicity), diagnosis, vaccine development and response to antiviral therapy (Zein, 2000; Legrand-Abravanel et al., 2009). Characterizing HCV genetic diversity may provide valuable insights into diagnosis, prevention and therapy of HCV infection. One important finding of this study is the identification of the new HCV subtype 6xg, which increases the subtype number of the genotype 6 to 31 (6a-6xh) together with another new subtype 6xh (Wu et al., 2018).

Currently, eight HCV genotypes and 86 subtypes were classified (Smith et al., 2014; Borgia et al., 2018). Different HCV genotypes displayed distinct epidemiological profiles (Gower et al., 2014; Messina et al., 2015). The genotype 1 is globally epidemic, while the others are generally restricted to specific geographical regions: the genotypes 2 and 3 are primarily prevalent in Asia and Europe, the genotypes 4 and 5 in Africa, and the genotype 6 appears to be restricted to Southeast Asia (Gower et al., 2014; Messina et al., 2015). Relative to other HCV genotypes, the genotype 6 is more diverse and has diverged into multiple different subtypes, including 31 confirmed subtypes, as well as some unassigned variants (Smith et al., 2014; Thong et al., 2014; Wu et al., 2018). As a new subtype, HCV 6xg is more genetically related to the subtypes 6n and 6xe (Figure 3A) and is estimated to have originated during 1984–1988 (Figure 5). Although the three representative full-length genomic sequences were isolated from IDUs in Kachin, Myanmar, it remains difficult to determine where the subtype 6xg arose because more 6xg sequences were found among IDUs in Dehong prefecture of Yunnan (Figure 4). In spite of this, the appearance of this new subtype among IDUs in the China–Myanmar border region could have significant epidemiological and public health implications.

Hepatitis C virus genotype 6 was mainly circulating in southwest China and other Southeast Asian countries (e.g., Myanmar, Vietnam, Thailand, and Laos) (Gower et al., 2014; Thong et al., 2014; Messina et al., 2015). Although 31 HCV 6 subtypes had been identified, only few subtypes (e.g., 6a, 6n, 6m, 6xa, and 6xg) caused epidemics (Thong et al., 2014; Wu et al., 2018). The subtype 6n was prevalent in China, Myanmar and other Southeast Asian countries (e.g., Thailand, Malaysia), while the subtype 6a was mainly circulating among IDUs in the south part of China, including Yunnan (Zhang et al., 2011, 2013; Chen et al., 2015; Qi et al., 2016; Wan et al., 2016), and the subtype 6m was mainly found in blood donors of Myanmar and Thailand (Shinji et al., 2004; Jutavijittum et al., 2009; Wasitthankasem et al., 2015). Interestingly, the prevalence of subtypes 6xa and 6xg were restricted to the China–Myanmar border region (Lwin et al., 2007; Zhang et al., 2013; Chen et al., 2015; Win et al., 2016). As a newly identified subtype, 6xg accounted for 12.0% (3/25) of all strains sampled in Kachin, and 6.0% (15/249) in Dehong prefecture, indicating that 6xg has become the main genotype 6 strains circulating in the China–Myanmar border region after the subtypes 6n and 6xa (Figure 1). The rapidly increasing prevalence of the 6xg strains occurred during 2005–2009 as reflected by the population growth curve (Figure 6). It is unclear if the increasing prevalence of this strain in the region represents a high transmissibility of the new subtype 6xg or circulation within a susceptible high risk group. The subtype 6xg diverged from the subtype 6n (Figure 3A). It is clear, however, that compared to 6n and other subtypes of the genotype 6, 6xg had a one-amino acid insertion (Lys at position 360) in the RRKR/K motif of NS5A, which changes this motif to RRKKR/K (Figure 3B). NS5A is a zinc-containing phosphoprotein involved in multiple and diverse functions in HCV replication, interferon resistance, and pathogenesis (Tellinghuisen et al., 2005; He et al., 2006), and the RRKR motif serves as a specific cleavage site for the furin family of intracellular proteinases (Pei and Weiss, 1995). Whether and how the Lys insertion influences the function of NS5A need to be experimentally investigated.

The China–Myanmar border region was the worst-hit region by HCV and HIV-1, where IDU was the most high-risk behavior for infection with both viruses (Zhang et al., 2002; Zhou et al., 2011, 2012; Li et al., 2014). HCV genetic diversity among IDUs in Yunnan had been extensively studied (Xia et al., 2008; Zhang et al., 2013; Chen et al., 2015; Wan et al., 2016), and three HCV genotypes (1, 3, and 6), including nine subtypes (1a, 1b, 3a, 3b, 6a, 6n, 6v, 6xa, and 6xg), were reported to be prevalent among IDUs in Yunnan (Figure 1). However, HCV genetic diversity among IDUs in Myanmar was less concerned in spite of high HCV prevalence among this cohort. In this study, we characterized HCV genetic diversity among IDUs in Kachin, Myanmar (Figure 1). Three HCV genotypes (1, 3, and 6), including six subtypes (1a, 3a, 3b, 6n, 6xa, and 6xg), as well as an unassigned genotype 6 variant, were detected among this cohort. The subtype distribution among IDUs was similar to that among IDUs in Dehong, another side of the border, but very distinct from those among general population (e.g., blood donors) in Myanmar and among IDUs in other regions of Yunnan (e.g., Kunming and Honghe) (Figure 1). The main difference was that the subtypes 6xg and 6xa appeared to be mainly prevalent among IDUs in the China–Myanmar border region. In contrast, there were rare strains from the subtypes 6a and 6m in this region, which had high prevalence in other regions of Yunnan and Myanmar, respectively (Figure 1). Furthermore, the most common HCV subtype 1b was also rarely detected among IDUs in this region. These results suggest that the China–Myanmar border region had a unique pattern of HCV genetic diversity among IDUs.

In China, pegylated interferon (PEG-IFN) plus ribavirin (RBV) (PEG-IFN+RBV) are recommended as a pan-genotypic regimen for HCV infection (Wei and Hou, 2015; Chen et al., 2018). The combination of sofosbuvir (SOF) and daclatasvir (DCV) is recommended in Myanmar (Ministry of Health and Sports Myanmar, 2017). Different direct-acting antiviral (DAA) therapies appeared to have various sustained virologic response (SVR) for different HCV genotypes. A systematic review and meta-analysis revealed that SVR of the PEG-IFN+RBV regimen to HCV genotype 6 was similar to genotypes 2 and 3, but higher than genotype 1 (Thong et al., 2015). The safety and efficacy of SOF-based DAA regimens for treatment of HCV genotype 6 were well demonstrated by some studies conducted in China and the United States (Wong et al., 2017; Hu et al., 2018; Wu et al., 2019). In particular, no significant difference in efficacy of SOF-based DAA regimens was observed between genotype 6 and genotypes 1-4, except for the combination of SOF and ledipasvir (LDV), which had lower SVR for genotype 6 than genotype 1 (Horner and Naggie, 2015; Hlaing et al., 2017; Fan et al., 2018; Hu et al., 2018). Furthermore, there was no study to compare the efficacy of various DAA regimens on different subtypes or variants of genotype 6.

Early drug trafficking and current labor migration from Myanmar to Yunnan driven by rapid economic development in China facilitate the cross-border transmissions of IDU-associated infectious diseases (e.g., HIV-1 and HCV) in the China–Myanmar border region (Beyrer et al., 2000; Liu et al., 2012; Chen et al., 2015, 2018b). As a China–Myanmar border prefecture, Dehong serves as an important “hub” not only linking China and Myanmar, but also mediating viral transmission (Chen et al., 2015, 2018a,b). Currently, Dehong has a large number of Burmese (including IDUs) who live, work or stay here. Increasing evidences suggested that Burmese IDUs staying in Yunnan played a crucial role in the cross-border transmission of IDU-associated viruses in spite that they were separated from the local IDUs to some extent (Wan et al., 2016; Chen et al., 2018a,b). Apart from the three strains identified in Kachin, Myanmar in this study, most other 6xg strains were found among Burmese IDUs staying in Dehong prefecture, and two strains were detected from Chinese IDUs, supporting a transmission link between Burmese and Chinese IDUs. Importantly, the fact of many 6xg-carring Burmese IDUs staying in Dehong will increase the possibility of 6xg causing epidemic outbreak among Chinese IDUs. The same concern was from HCV 6xa, which originated earlier in Myanmar (data not shown), and had caused epidemic outbreak among IDUs in both sides of the China-Myanmar border and sporadic outbreak in other regions of China (Qi et al., 2016; Wan et al., 2016). Given previous epidemiologic and transmission patterns of HIV-1, it is likely that HCV 6xg and 6xa spread from the border region to other regions of both China and Myanmar, and even across Southeast Asia. Therefore, closely monitoring the molecular epidemiology of HCV genotype 6 in this region and strengthening the management of Burmese IDUs staying in Yunnan are highly encouraged.

This study has several limitations. Frist, HCV exists in a quasispecies form in human body (Martell et al., 1992). It will be better to obtain a single full-length genome sequence from a single virus. However, because of failure in amplification of near full-length HCV genome (>5000 bps), we amplified and sequenced 10 overlapping HCV genomic segments to obtain the whole genome sequence. Therefore, the genomic sequences obtained in this study contain some ambiguous (or degenerate) nucleotides (quasispecies population). Second, previous studies showed that vast majority of IDUs in Yunnan, especially in the China-Myanmar border area, were male (Zhou et al., 2011, 2012; Li et al., 2014; Chen et al., 2018b). Similarly, only few female IDUs’ samples were available in this study. Although no significant difference in HCV positive rate was observed between male and female IDUs (Table 1), we were unable to determine whether the gender has an effect on demographic characteristics and HCV subtype distribution due to relatively small sample size of female IDUs. Third, in addition to the identification of HCV 6xg, we found that the strain KS88 might also be a potential new subtype of HCV genotype 6. Because only one sample was found to carry this strain, which did not meet the criterion that the virus strain of a new subtype must be identified in at least three epidemiologically unlinked individuals, we did not perform further analysis of this strain. Whether the strain KS88 is a new HCV subtype needs to be determined by expanding the sample size in future study.

In summary, we firstly identified a new HCV subtype 6xg, and reported the molecular epidemiological characteristics of HCV among IDUs in Kachin, Myanmar. The appearance of 6xg and the unique HCV subtype profile in the China–Myanmar border region have significant epidemiological and public health implications.

## Ethics Statement

This is to confirm that the research protocol of the study entitled “Identification of a new HCV subtype 6xg among injection drug users in Kachin, Myanmar” (Principal investigator: Y-TZ), which has been reviewed and approved by the internal review board of Kunming Institute of Zoology, Chinese Academy of Sciences (approval number: SWYX-2013023; approval date: September 6, 2013).

## Author Contributions

Y-TZ, CZ, and MY contributed to conception, designed the study, and interpreted the results. LD and XC collected the samples. MY and YW performed the experiments. MY analyzed the data. Y-TZ, CZ, and MY wrote the manuscript. Y-TZ supervised the study. All authors read the manuscript and approved the submitted version.

## Conflict of Interest Statement

The authors declare that the research was conducted in the absence of any commercial or financial relationships that could be construed as a potential conflict of interest.

## Footnotes

1 https://talk.ictvonline.org/ictv_wikis/flaviviridae/w/Sg_flavi/56/hcv-classification

2 https://hcv.lanl.gov/content/sequence/VIRALIGN/viralign.html
