# Supplementary material for: Identification of a New HCV Subtype 6xg Among Injection Drug Users in Kachin, Myanmar
Source: Front Microbiol. 2019 Apr 18;10:814. doi: 10.3389/fmicb.2019.00814 (PMC6482298; doi:10.3389/fmicb.2019.00814)
Supplement: Supplementary file 1 [file Table_1.DOCX]

**Table S1. List of primers used in this study.**

| **Application** | **Location** | **Name** | **Polarity** | **Sequence (5’→ 3’)** | **Position (H77 AF_009606)** | **Reference** |
| --- | --- | --- | --- | --- | --- | --- |
| **Genotyping** | C/E2 | C/E2-F1 | Forward | GCCGACCTCATGGGGTACAT | 732-751 | (Wan et al., 2016) |
|  |  | C/E2-R1 | Reverse | ARTTBTYDGTRCANGGRTARTGCCA | 2187-2211 | (Wan et al., 2016) |
|  |  | C/E2-F2 | Forward | CCYGGTTGCTCYTTYTCTATCTT | 849-871 | (Wan et al., 2016) |
|  |  | C/E2-R2 | Reverse | GTNADCCANGGNCCNGMNCCRCA | 2130-2152 | (Wan et al., 2016) |
|  | NS5B | NS5B-F1 | Forward | GGSTTYTCNTATGAYACCMGVTGYTTTGA | 8247-8275 | (Wan et al., 2016) |
|  |  | NS5B-R1 | Reverse | CTACCCCTACNGHDAGTAGGAGTAGGC | 9325-9351 | (Wan et al., 2016) |
|  |  | NS5B-F2 | Forward | GCTGYTTTGAYTCAACNGTCAC | 8266-8287 | (Wan et al., 2016) |
|  |  | NS5B-R2 | Reverse | GRGCHYGVGACACGCTGTGATANATGTC | 9276-9303 | (Wan et al., 2016) |
| **Amplification of near full-length HCV genome** | 1 | 5-END | Forward | GCCAGCCCCTAAYGGGGCGA | 1-20 | (Lu et al., 2007) |
|  |  | 346R | Reverse | GTGCTCATGVTGCACGGTCTACGAGACCT | 346-318R | (Lu et al., 2007) |
|  |  | HCV1 | Forward | GGCGACACTCCACCATGAATCACT | 18-41 | (Lu et al., 2005) |
|  |  | 302R | Reverse | AGCACCCTATCAGGCAGTACCACAAGGCCT | 302-273R | (Lu et al., 2007) |
|  | 2 | HCV1 | Forward | GGCGACACTCCACCATGAATCACT | 18-41 | (Lu et al., 2005) |
|  |  | C/E2-R1 | Reverse | ARTTBTYDGTRCANGGRTARTGCCA | 2187-2211 | (Wan et al., 2016) |
|  |  | HCV2 | Forward | AATCACTCCCCTGTGAGGAACTACTGT | 35-61 | (Lu et al., 2005) |
|  |  | C/E2-R2 | Reverse | GTNADCCANGGNCCNGMNCCRCA | 2130-2152 | (Wan et al., 2016) |
|  | 3 | SF1 | Forward | GTGTCYTGAACGCATGTCYTCCT | 1696-1717 | This study |
|  |  | SR1 | Reverse | CAGTRACAATAGCAAGTGCRAGAAA | 2803-2827 | This study |
|  |  | 86-1749 | Forward | CCTATCACATATGCCAACACATCC | 1749-1772 | (Lu et al., 2007) |
|  |  | 6m-2498R | Reverse | GTAYTCCCACTTSAYWGCCCACGA | 2472-2495 | (Lu et al., 2007) |
|  | 4 | 86-2305 F | Forward | ACGACAGGGACAGAGTGGAGATGA | 2305-2328 | (Lu et al., 2007) |
|  |  | 86-3670R | Reverse | CCTACCAGGTCTTGGTCGACATTG | 3647-3670 | (Lu et al., 2007) |
|  |  | 86-2334 F | Forward | CTTCTGTTCTCCACGACAGAGTTG | 2334-2357 | (Lu et al., 2007) |
|  |  | 86-3651R | Reverse | CATTGGTGTACATCTGACACACAG | 3628-3651 | (Lu et al., 2007) |
|  | 5 | 86-3542 F | Forward | GACCTTCCTCGCTACCTCTGTCAA | 3542-3565 | (Lu et al., 2007) |
|  |  | HCV8 R | Reverse | TGGTGAAGGTAGGGTCAAGGCTGAAAT | 4729-4755 | (Lu et al., 2005) |
|  |  | 86-3570 F | Forward | GTTCTTTGGACGGTTTACCATGGC | 3570-3593 | (Lu et al., 2007) |
|  |  | HCV9 R | Reverse | GTATAGCCGGTCATGAGGGCATC | 4653-4675 | (Lu et al., 2005) |
|  | 6 | 6v-4282 | Forward | TAYGAYRTCATYATWTGYGAYGAGTG | 4269-4294 | (Wang et al., 2009) |
|  |  | 6v-5956R | Reverse | GGRGYCTCGCCGCTCATGATYTTAAA | 5937-5962 | (Wang et al., 2009) |
|  |  | 6v-4292 | Forward | AYRTCATYATWTGYGAYGAGTGYCAYTC | 4273-4300 | (Wang et al., 2009) |
|  |  | 3-5681R | Reverse | TGRATCCCRCTSACAAARTTCCACAT | 5634-5659 | (Lu et al., 2013) |
|  | 7 | 86-5464 F | Forward | TGGAGGAATGCTCTAAACACATCC | 5464-5487 | (Lu et al., 2007) |
|  |  | 86-7495R | Reverse | GAGAATGTCTCGACGTCGCCTTTG | 7472-7495 | (Lu et al., 2007) |
|  |  | 86-5504 F | Forward | CCAGCTCATAGCAGAGCAGTTCAA | 5504-5527 | (Lu et al., 2007) |
|  |  | 86-7468R | Reverse | TCAGCAGAACCGCTATCATTGACT | 7445-7468 | (Lu et al., 2007) |
|  | 7^a^ | 6v-5464 | Forward | TATCAGCARTAYGAYGAGATGGARGARTG | 5445-5473 | (Wang et al., 2009) |
|  |  | 86-7495R | Reverse | GAGAATGTCTCGACGTCGCCTTTG | 7472-7495 | (Lu et al., 2007) |
|  |  | 6v-5482 | Forward | ATGGAGGARTGYTCWMGRCACMTCCCYTA | 5463-5491 | (Wang et al., 2009) |
|  |  | 86-7468R | Reverse | TCAGCAGAACCGCTATCATTGACT | 7445-7468 | (Lu et al., 2007) |
|  | 8 | SF2 | Forward | CGAGTATGATGACAGGGAGCCA | 7128-7149 | This study |
|  |  | SR2 | Reverse | GTCAAAGTTRGTGAGGCCTGCTG | 8528-8550 | This study |
|  |  | 86-7380 F | Forward | CAGCTTGCTGAGAAGTCGTTTCCA | 7380-7403 | (Lu et al., 2007) |
|  |  | 6n-8379R | Reverse | AGATGTTATGGCCTTCCTGGCTTC | 8356-8379 | (Lu et al., 2006) |
|  | 9 | NS5B-F1 | Forward | GGSTTYTCNTATGAYACCMGVTGYTTTGA | 8247-8275 | (Wan et al., 2016) |
|  |  | NS5B-R1 | Reverse | CTACCCCTACNGHDAGTAGGAGTAGGC | 9325-9351 | (Wan et al., 2016) |
|  |  | NS5B-F2 | Forward | GCTGYTTTGAYTCAACNGTCAC | 8266-8287 | (Wan et al., 2016) |
|  |  | NS5B-R2 | Reverse | GRGCHYGVGACACGCTGTGATANATGTC | 9276-9303 | (Wan et al., 2016) |
|  | 10 | K46-9135 | Forward | CCGCTATCTGCGGTAAGTACCTCT | 9135-9158 | (Wang et al., 2009) |
|  |  | 7er | Reverse | GGGGAGCAGGTAGATGCCTA | 9349-9368 | (Tremeaux et al., 2016) |
|  |  | 86-9219F2 | Forward | GCDATWTGCGGCAAGTACCTCTT | 9165-9187 | (Lu et al., 2007) |
|  |  | 9353R | Reverse | RGSDGGKACHARRAAGATGCCTACCCCTAC | 9342-9371 | (Lu et al., 2006) |
|  | Reverse transcription | 3UTR1 | Reverse | GACCTTTCACAGCTAGCCGTGACT | 9569-9592 | (Tremeaux et al., 2016) |
|  |  | 7er | Reverse | GGGGAGCAGGTAGATGCCTA | 9349-9368 | (Tremeaux et al., 2016) |
|  |  | 9353R | Reverse | RGSDGGKACHARRAAGATGCCTACCCCTAC | 9342-9371 | (Lu et al., 2006) |
|  |  | A5376 R | Reverse | GACCCTGCCCACTATGACCACGCA | 5376-5399 | (Lu et al., 2005) |

**References**

Lu, L., Li, C., Fu, Y., Thaikruea, L., Thongswat, S., Maneekarn, N., et al. (2007). Complete genomes for hepatitis C virus subtypes 6f, 6i, 6j and 6m: viral genetic diversity among Thai blood donors and infected spouses. *J Gen Virol* 88**,** 1505-1518. doi: 10.1099/vir.0.82604-0.

Lu, L., Li, C., Yuan, J., Lu, T., Okamoto, H., and Murphy, D.G. (2013). Full-length genome sequences of five hepatitis C virus isolates representing subtypes 3g, 3h, 3i and 3k, and a unique genotype 3 variant. *J Gen Virol* 94**,** 543-548. doi: 10.1099/vir.0.049668-0.

Lu, L., Nakano, T., Li, C., Fu, Y., Miller, S., Kuiken, C., et al. (2006). Hepatitis C virus complete genome sequences identified from China representing subtypes 6k and 6n and a novel, as yet unassigned subtype within genotype 6. *J Gen Virol* 87**,** 629-634. doi: 10.1099/vir.0.81400-0.

Lu, L., Nakano, T., Smallwood, G.A., Heffron, T.G., Robertson, B.H., and Hagedorn, C.H. (2005). A refined long RT-PCR technique to amplify complete viral RNA genome sequences from clinical samples: application to a novel hepatitis C virus variant of genotype 6. *J Virol Methods* 126**,** 139-148. doi: 10.1016/j.jviromet.2005.01.031.

Tremeaux, P., Caporossi, A., Ramiere, C., Santoni, E., Tarbouriech, N., Thelu, M.A., et al. (2016). Amplification and pyrosequencing of near-full-length hepatitis C virus for typing and monitoring antiviral resistant strains. *Clin Microbiol Infect* 22**,** 460.e461-460.e410. doi: 10.1016/j.cmi.2016.01.015.

Wan, Z., Chen, Q., Chen, X., Duo, L., Li, P., Zheng, Y.T., et al. (2016). HCV Diversity among Chinese and Burmese IDUs in Dehong, Yunnan, China. *PLoS One* 11**,** e0163062. doi: 10.1371/journal.pone.0163062.

Wang, Y., Xia, X., Li, C., Maneekarn, N., Xia, W., Zhao, W., et al. (2009). A new HCV genotype 6 subtype designated 6v was confirmed with three complete genome sequences. *J Clin Virol* 44**,** 195-199. doi: 10.1016/j.jcv.2008.12.009.

**Table S2. The number of nucleotides/amino acids in each genomic region.**

| Isolate | Genome | ORF | 5’UTR | Core | E1 | E2 | P7 | NS2 | NS3 | NS4A | NS4B | NS5A | NS5B | 3UTR |
| --- | --- | --- | --- | --- | --- | --- | --- | --- | --- | --- | --- | --- | --- | --- |
| 6n_DQ278894 | 9440 | 9046/3016 | 341 | 573/191 | 576/192 | 1092/364 | 189/63 | 651/217 | 1893/631 | 162/54 | 783/261 | 1353/451 | 1774/591 | 53 |
| KS27 | 9318 | 9046/3015 | 272 | 573/191 | 576/192 | 1092/364 | 189/63 | 651/217 | 1893/631 | 162/54 | 783/261 | 1356/452 | 1771/590 | - |
| KS81 | 9318 | 9046/3015 | 272 | 573/191 | 576/192 | 1092/364 | 189/63 | 651/217 | 1893/631 | 162/54 | 783/261 | 1356/452 | 1771/590 | - |
| KS86 | 9318 | 9046/3015 | 272 | 573/191 | 576/192 | 1092/364 | 189/63 | 651/217 | 1893/631 | 162/54 | 783/261 | 1356/452 | 1771/590 | - |
| HCV reference:  H77 | 9646 | 9036/3011 | 341 | 573/191 | 576/192 | 1089/363 | 189/63 | 651/217 | 1893/631 | 162/54 | 783/261 | 1344/448 | 1776/591 | 269 |

**Table S3. Pairwise comparisons of amino acid and nucleotide similarities of 3 HCV 6xg strains and 29 reference genotype 6 sequences.**

| **Subtypes** | **6a** | **6b** | **6c** | **6d** | **6e** | **6f** | **6g** | **6h** | **6i** | **6j** | **6k** | **6l** | **6m** | **6n** | **6o** | **6p** | **6q** | **6r** | **6s** | **6t** | **6u** | **6v** | **6w** | **6xa** | **6xb** | **6xc** | **6xd** | **6xe** | **6xf** | **KS27** | **KS81** | **KS86** |
| --- | --- | --- | --- | --- | --- | --- | --- | --- | --- | --- | --- | --- | --- | --- | --- | --- | --- | --- | --- | --- | --- | --- | --- | --- | --- | --- | --- | --- | --- | --- | --- | --- |
| **6a** |  | **86.5** | **79.3** | **79.8** | **79.6** | **79.8** | **78.8** | **79.4** | **78.8** | **77.5** | **79.7** | **78.5** | **78.2** | **79.3** | **78.4** | **79.4** | **76.6** | **79.4** | **78.0** | **79.2** | **79.1** | **78.9** | **78.7** | **79.8** | **78.6** | **78.8** | **86.0** | **79.6** | **78.5** | **79.8** | **79.7** | **79.9** |
| **6b** | **79.1** |  | **81.3** | **81.5** | **81.4** | **81.0** | **80.5** | **80.9** | **80.7** | **79.6** | **81.1** | **79.7** | **79.2** | **81.4** | **79.9** | **81.1** | **78.0** | **81.0** | **80.0** | **81.2** | **80.9** | **79.9** | **79.5** | **81.4** | **79.8** | **80.4** | **89.9** | **81.3** | **80.3** | **81.3** | **81.2** | **81.2** |
| **6c** | **71.9** | **72.8** |  | **86.7** | **85.6** | **84.5** | **81.6** | **80.3** | **80.6** | **79.2** | **82.5** | **80.5** | **79.9** | **81.7** | **83.6** | **85.2** | **82.1** | **84.8** | **81.1** | **85.3** | **84.9** | **80.1** | **80.4** | **81.2** | **80.6** | **85.1** | **80.0** | **81.9** | **83.4** | **81.8** | **81.6** | **81.3** |
| **6d** | **71.5** | **72.6** | **78.6** |  | **85.8** | **84.8** | **82.6** | **81.2** | **80.4** | **79.9** | **82.4** | **80.7** | **80.9** | **82.3** | **84.2** | **85.2** | **82.0** | **85.1** | **82.3** | **85.5** | **85.3** | **80.6** | **81.1** | **82.5** | **80.7** | **85.1** | **80.9** | **82.5** | **85.0** | **82.5** | **82.4** | **82.6** |
| **6e** | **71.4** | **72.3** | **77.0** | **76.8** |  | **85.3** | **82.0** | **81.2** | **81.4** | **79.8** | **82.7** | **81.0** | **80.6** | **82.0** | **85.0** | **85.9** | **83.7** | **85.4** | **82.3** | **86.3** | **90.3** | **80.2** | **81.5** | **82.4** | **80.9** | **88.7** | **80.6** | **82.1** | **84.8** | **82.2** | **82.1** | **82.2** |
| **6f** | **71.7** | **72.3** | **76.9** | **76.8** | **76.4** |  | **82.2** | **80.9** | **80.4** | **80.0** | **82.2** | **81.3** | **80.3** | **81.7** | **83.9** | **84.4** | **82.1** | **89.9** | **82.0** | **84.9** | **85.0** | **80.5** | **80.3** | **81.5** | **80.6** | **83.9** | **80.8** | **82.2** | **83.4** | **82.2** | **82.1** | **82.2** |
| **6g** | **71.7** | **72.5** | **74.2** | **74.2** | **74.2** | **74.2** |  | **80.6** | **79.8** | **78.7** | **81.1** | **80.0** | **79.7** | **80.7** | **80.4** | **80.9** | **78.7** | **82.2** | **79.6** | **81.5** | **81.9** | **79.6** | **83.0** | **80.5** | **80.1** | **80.9** | **80.1** | **81.9** | **80.9** | **81.3** | **81.2** | **80.7** |
| **6h** | **71.8** | **72.5** | **72.8** | **73.2** | **72.8** | **73.1** | **72.6** |  | **86.7** | **86.2** | **86.1** | **84.3** | **83.9** | **85.9** | **80.3** | **81.4** | **78.5** | **80.9** | **79.9** | **81.2** | **81.1** | **81.0** | **79.8** | **81.5** | **84.6** | **81.0** | **80.0** | **85.4** | **80.4** | **85.9** | **85.6** | **85.6** |
| **6i** | **71.8** | **72.2** | **73.6** | **73.1** | **73.2** | **73.0** | **72.3** | **78.6** |  | **88.1** | **84.4** | **83.3** | **82.5** | **83.9** | **80.1** | **80.6** | **78.3** | **80.5** | **78.8** | **80.8** | **81.0** | **79.6** | **79.9** | **80.4** | **82.5** | **79.9** | **79.6** | **83.9** | **80.0** | **84.0** | **83.8** | **83.7** |
| **6j** | **71.2** | **72.0** | **72.7** | **72.9** | **72.7** | **73.2** | **72.2** | **78.8** | **82.0** |  | **83.4** | **82.8** | **82.0** | **83.7** | **78.4** | **79.5** | **77.6** | **80.1** | **78.5** | **79.6** | **79.7** | **78.9** | **78.1** | **79.7** | **82.3** | **78.5** | **78.8** | **83.9** | **79.3** | **83.4** | **83.2** | **83.6** |
| **6k** | **72.2** | **72.9** | **74.0** | **73.4** | **73.3** | **73.3** | **72.8** | **77.0** | **76.7** | **76.1** |  | **87.9** | **85.3** | **87.3** | **81.4** | **82.8** | **79.5** | **82.3** | **80.9** | **82.7** | **81.6** | **81.7** | **80.5** | **82.5** | **89.6** | **81.6** | **80.6** | **87.3** | **80.8** | **87.4** | **87.2** | **87.2** |
| **6l** | **72.2** | **72.2** | **73.6** | **73.5** | **73.2** | **73.6** | **73.1** | **76.6** | **76.5** | **76.4** | **80.2** |  | **83.1** | **85.7** | **80.0** | **80.8** | **77.8** | **81.2** | **78.9** | **81.0** | **80.6** | **80.1** | **79.2** | **81.3** | **85.6** | **79.8** | **79.0** | **85.8** | **79.5** | **85.8** | **85.5** | **85.6** |
| **6m** | **71.9** | **72.3** | **73.4** | **73.5** | **72.8** | **73.3** | **72.4** | **76.7** | **76.7** | **76.0** | **78.2** | **77.8** |  | **86.9** | **79.6** | **81.1** | **78.0** | **80.5** | **78.5** | **80.9** | **80.0** | **79.8** | **79.0** | **81.1** | **83.2** | **80.0** | **79.1** | **86.9** | **80.2** | **87.5** | **87.2** | **87.4** |
| **6n** | **71.8** | **72.7** | **73.8** | **74.1** | **73.4** | **73.8** | **72.9** | **77.5** | **77.0** | **76.9** | **78.3** | **78.6** | **80.8** |  | **81.3** | **82.2** | **79.3** | **82.1** | **80.6** | **82.0** | **81.3** | **81.3** | **80.3** | **82.4** | **85.1** | **81.3** | **80.4** | **91.3** | **80.9** | **91.9** | **91.7** | **91.5** |
| **6o** | **72.0** | **72.6** | **77.2** | **76.8** | **77.3** | **76.7** | **73.5** | **73.1** | **73.1** | **72.5** | **72.9** | **73.1** | **73.4** | **73.6** |  | **85.3** | **81.8** | **83.5** | **81.5** | **84.4** | **84.8** | **79.8** | **79.8** | **81.1** | **79.9** | **83.9** | **79.3** | **81.2** | **82.4** | **81.1** | **81.1** | **81.2** |
| **6p** | **72.4** | **72.9** | **77.7** | **77.1** | **77.5** | **76.3** | **73.7** | **73.1** | **73.7** | **72.8** | **74.0** | **73.6** | **73.7** | **74.3** | **79.2** |  | **82.2** | **84.5** | **82.0** | **85.4** | **85.0** | **80.0** | **80.9** | **81.7** | **80.9** | **85.1** | **80.4** | **82.6** | **83.0** | **82.7** | **82.5** | **82.5** |
| **6q** | **71.4** | **71.8** | **76.6** | **76.2** | **76.8** | **76.2** | **73.3** | **72.8** | **73.2** | **72.8** | **73.3** | **73.3** | **73.1** | **73.8** | **76.4** | **76.8** |  | **81.4** | **79.3** | **82.8** | **83.1** | **77.8** | **78.3** | **78.3** | **77.5** | **82.9** | **77.7** | **79.6** | **80.5** | **79.2** | **79.1** | **79.1** |
| **6r** | **71.9** | **72.8** | **77.0** | **77.3** | **75.9** | **82.4** | **73.7** | **73.0** | **73.0** | **72.7** | **73.3** | **73.5** | **73.4** | **74.2** | **76.2** | **76.7** | **76.4** |  | **81.4** | **84.9** | **84.0** | **80.2** | **80.6** | **81.9** | **80.9** | **83.7** | **80.8** | **82.5** | **83.8** | **82.5** | **82.3** | **82.3** |
| **6s** | **71.2** | **71.9** | **73.5** | **74.4** | **74.3** | **74.1** | **72.7** | **72.4** | **72.4** | **72.2** | **72.9** | **72.9** | **72.4** | **72.9** | **74.4** | **73.9** | **73.7** | **74.5** |  | **82.0** | **81.7** | **78.6** | **78.5** | **80.3** | **79.4** | **82.2** | **78.8** | **81.0** | **80.7** | **80.5** | **80.4** | **80.4** |
| **6t** | **71.2** | **72.4** | **77.1** | **77.3** | **77.1** | **76.9** | **73.6** | **73.3** | **73.4** | **73.0** | **73.6** | **73.5** | **73.4** | **73.7** | **77.8** | **78.0** | **77.4** | **76.9** | **74.4** |  | **85.6** | **80.5** | **80.8** | **82.2** | **80.6** | **85.4** | **80.5** | **82.3** | **83.6** | **82.3** | **82.2** | **82.0** |
| **6u** | **71.3** | **72.3** | **76.8** | **77.3** | **83.0** | **76.5** | **74.1** | **72.5** | **73.0** | **72.9** | **72.6** | **73.3** | **72.6** | **73.4** | **76.3** | **77.3** | **77.0** | **75.7** | **73.7** | **77.3** |  | **80.1** | **81.7** | **81.2** | **80.0** | **87.6** | **80.0** | **81.9** | **83.3** | **81.8** | **81.7** | **81.9** |
| **6v** | **71.8** | **72.3** | **72.2** | **72.2** | **71.7** | **72.3** | **72.0** | **72.7** | **72.2** | **72.1** | **73.2** | **73.0** | **72.9** | **73.0** | **72.6** | **72.1** | **72.3** | **72.2** | **71.5** | **72.9** | **71.6** |  | **79.6** | **83.1** | **79.3** | **79.2** | **79.3** | **81.7** | **79.9** | **81.4** | **81.3** | **81.4** |
| **6w** | **71.4** | **72.2** | **73.3** | **73.1** | **73.6** | **73.6** | **75.1** | **72.3** | **72.5** | **72.0** | **72.6** | **72.5** | **72.3** | **72.8** | **73.4** | **73.3** | **73.4** | **73.6** | **72.7** | **73.8** | **73.9** | **71.8** |  | **79.8** | **78.7** | **80.6** | **78.7** | **80.5** | **79.5** | **80.2** | **80.2** | **79.9** |
| **6xa** | **72.3** | **72.8** | **73.9** | **74.4** | **74.2** | **73.9** | **72.7** | **73.1** | **73.1** | **73.3** | **74.4** | **73.8** | **73.9** | **74.1** | **73.8** | **73.6** | **72.5** | **73.7** | **73.1** | **73.7** | **73.3** | **74.6** | **72.5** |  | **81.2** | **80.8** | **79.9** | **82.4** | **80.8** | **82.8** | **82.6** | **83.1** |
| **6xb** | **71.4** | **71.9** | **73.3** | **73.6** | **73.0** | **73.6** | **72.9** | **76.3** | **76.0** | **75.8** | **82.4** | **79.8** | **77.2** | **77.5** | **72.6** | **73.1** | **72.9** | **73.0** | **72.6** | **73.3** | **72.5** | **72.4** | **72.3** | **74.0** |  | **79.9** | **79.0** | **85.0** | **79.5** | **85.0** | **84.8** | **84.7** |
| **6xc** | **71.8** | **72.0** | **76.6** | **76.5** | **80.4** | **76.0** | **73.7** | **72.4** | **72.8** | **72.5** | **72.7** | **72.9** | **73.0** | **73.6** | **77.0** | **77.3** | **77.3** | **75.9** | **74.3** | **77.2** | **80.6** | **71.2** | **73.2** | **73.1** | **72.5** |  | **79.2** | **81.6** | **83.1** | **81.6** | **81.4** | **81.3** |
| **6xd** | **79.5** | **84.5** | **72.3** | **72.2** | **71.7** | **73.0** | **72.1** | **72.1** | **72.0** | **72.2** | **72.8** | **72.5** | **72.1** | **72.3** | **72.7** | **72.9** | **71.7** | **72.7** | **72.2** | **72.2** | **72.3** | **71.6** | **72.0** | **72.7** | **72.0** | **72.0** |  | **80.2** | **79.5** | **80.6** | **80.4** | **80.6** |
| **6xe** | **71.7** | **72.6** | **73.9** | **73.8** | **73.0** | **73.5** | **73.0** | **76.7** | **77.1** | **76.6** | **78.5** | **78.6** | **80.5** | **84.6** | **73.5** | **73.8** | **73.5** | **73.5** | **72.6** | **73.4** | **73.2** | **72.6** | **72.8** | **73.7** | **77.1** | **73.1** | **72.8** |  | **82.0** | **91.7** | **91.6** | **91.2** |
| **6xf** | **71.3** | **72.1** | **75.7** | **76.8** | **76.4** | **75.0** | **73.1** | **72.7** | **73.2** | **73.0** | **72.8** | **73.2** | **73.4** | **73.1** | **75.5** | **76.1** | **75.3** | **75.5** | **73.4** | **76.1** | **76.1** | **72.2** | **72.9** | **73.1** | **72.6** | **76.0** | **72.1** | **73.8** |  | **81.6** | **81.3** | **81.1** |
| **KS27** | **71.9** | **72.7** | **74.1** | **74.1** | **73.2** | **73.6** | **73.2** | **76.7** | **76.7** | **76.7** | **78.3** | **78.3** | **80.5** | **85.6** | **73.7** | **74.3** | **73.7** | **74.1** | **72.7** | **73.8** | **73.6** | **73.2** | **73.1** | **74.7** | **77.3** | **73.5** | **72.7** | **85.2** | **73.5** |  | **98.5** | **96.6** |
| **KS81** | **72.0** | **72.7** | **74.0** | **74.0** | **73.2** | **73.6** | **73.2** | **76.6** | **76.7** | **76.7** | **78.2** | **78.3** | **80.4** | **85.6** | **73.7** | **74.2** | **73.6** | **74.1** | **72.7** | **73.7** | **73.5** | **73.2** | **73.1** | **74.7** | **77.2** | **73.4** | **72.6** | **85.2** | **73.3** | **99.3** |  | **96.9** |
| **KS86** | **71.8** | **72.5** | **73.8** | **73.8** | **73.2** | **73.6** | **72.7** | **76.8** | **76.5** | **76.5** | **77.9** | **78.4** | **80.5** | **85.3** | **73.7** | **74.0** | **73.5** | **74.0** | **72.6** | **73.7** | **73.4** | **73.0** | **72.9** | **74.6** | **77.2** | **73.2** | **72.7** | **84.9** | **73.2** | **96.1** | **96.2** |  |

Amino acid and nucleotide similarities in upper right and in lower left were compared based on open reading frame and the full length genome sequences, respectively. The GenBank accession numbers of genotype 6 reference sequences used in this table are as follows: 6a: Y12083, 6b: D84262, 6c: EF424629, 6d: D84263, 6e: DQ314805, 6f: DQ835760, 6g: D63822, 6h: D84265, 6i: DQ835762, 6j: DQ835761, 6k: D84264, 6l: EF424628, 6m: DQ835767, 6n: DQ278894, 6o: EF424627, 6p: EF424626, 6q: EF424625, 6r: EU408328, 6s: EU408329, 6t: EF632071, 6u: EU246940, 6v: EU158186, 6w: DQ278892, 6xa: EU408330, 6xb: JX183552, 6xc: KJ567651, 6xd: KM252789, 6xe: JX183557, 6xf: KJ567646.
